# 

**Published:** 1859-03

**Authors:** 


☞ *For Receipts, see 3d Page of Cover.*

### RECEIPTS FOR JOURNAL UP TO MARCH 30th, 1859.

### ILLINOIS.

| | | |
|---|---|---|
| Dr. V. Hurlbut | vol. 2, 1859, | $2 |
| J. D. Fisher | " " | 2 |
| H. W. Hopkins | " " | 2 |
| A. DeFoe | " " | 2 |
| J. H. Wiley | " " | 2 |
| F. D. Cass | " " | 2 |
| E. Ingalls | " " | 2 |
| J. H. Reeder | vol. 1, 1858, | 2 |
| S. E. Faircloth | " " | 2 |
| Hiram Hall | " " | 2 |
| John McHugh | " " | 2 |
| A. M. Catlin | vols. 6 & 1, 1858–9, | 4 |

### IOWA.

| | | |
|---|---|---|
| Wm. H. Fletcher | vol. 2, 1859, | 2 |
| H. Carpenter | vol. 1, 1858, | 2 |

### INDIANA.

| | | |
|---|---|---|
| N. Sherman | one-half vols. 1 & 2, 1858–9, | 3 |

### MINNESOTA.

| | | |
|---|---|---|
| J. H. Davis | vol. 2, 1859, | 2 |
| J. R. Dart | vols. 1 & half of 2, 1858–9, | 3 |

### MISSOURI.

| | | |
|---|---|---|
| C. M. Robertson | vol. 2, 1859, | 2 |

### ALABAMA.

| | | |
|---|---|---|
| C. McDonough | vol. 2, 1859, | 2 |

### CALIFORNIA.

| | | |
|---|---|---|
| C. A. Hathwell | vols. 1 & 2, 1858–9, | 4 |

